# Supplementary material for: Novel cell lines derived from Chinese hamster kidney tissue
Source: PLoS One. 2022 Mar 31;17(3):e0266061. doi: 10.1371/journal.pone.0266061 (PMC8970510; doi:10.1371/journal.pone.0266061)
Supplement: S1 Table — (DOCX) [file pone.0266061.s001.docx]

**S1 Table. Primers used for RT-PCR**

| **Genes** | **Sequence (5′→3′)** | |
| --- | --- | --- |
| *CD24* | FW | ACTGCTCCTACCCACGCAGA |
|  | RV | CGGGTCACACTGGACTTGGG |
| *CD133* | FW | GTGTTCCTGAGCGAAACCCC |
|  | RV | GCTGTGTGCTTCCATCACTTATG |
| *GAPDH* | FW | AACGTGTCCGTTGTGGATCTG |
|  | RV | GTCACTGTTGAAGTCGCAGG |
