# Supplementary material for: Novel cell lines derived from Chinese hamster kidney tissue
Source: PLoS One. 2022 Mar 31;17(3):e0266061. doi: 10.1371/journal.pone.0266061 (PMC8970510; doi:10.1371/journal.pone.0266061)
Supplement: S2 Table — (DOCX) [file pone.0266061.s002.docx]

**S2 Table. Karyotype analysis of pre-immortalized primary cells.**

| **Pr-CHK** | **Number of Chromosome [–]** | **Number of structurally normal chromosome [–]** | | | | | | | | | | | **Structurally abnormal chromosome** |
| --- | --- | --- | --- | --- | --- | --- | --- | --- | --- | --- | --- | --- | --- |
|  |  | **1** | **2** | **3** | **4** | **5** | **6** | **7** | **8** | **9** | **10** | **X** |  |
| #1 | 22 | 2 | 2 | 2 | 2 | 2 | 2 | 2 | 2 | 2 | 2 | 2 |  |
| #2 | 22 | 2 | 2 | 2 | 2 | 2 | 2 | 2 | 2 | 2 | 2 | 2 |  |
| #3 | 22 | 2 | 2 | 2 | 2 | 2 | 2 | 2 | 2 | 2 | 2 | 2 |  |
| #4 | 22 | 2 | 2 | 2 | 2 | 2 | 2 | 2 | 2 | 2 | 2 | 2 |  |
| #5 | 22 | 2 | 2 | 2 | 2 | 2 | 2 | 2 | 2 | 2 | 2 | 2 |  |
| #6^a^ | 22 | 2 | 2 | 2 | 2 | 2 | 2 | 2 | 2 | 2 | 2 | 2 |  |
| #7 | 22 | 2 | 2 | 2 | 2 | 2 | 2 | 2 | 2 | 2 | 2 | 2 |  |
| #8 | 22 | 2 | 2 | 2 | 2 | 2 | 2 | 2 | 2 | 2 | 2 | 2 |  |
| #9 | 22 | 2 | 2 | 2 | 2 | 2 | 2 | 2 | 2 | 2 | 2 | 2 |  |
| #10 | 22 | 2 | 2 | 2 | 2 | 2 | 2 | 2 | 2 | 2 | 2 | 2 |  |
| #11 | 22 | 2 | 2 | 2 | 2 | 2 | 2 | 2 | 2 | 2 | 2 | 2 |  |
| #12 | 22 | 2 | 2 | 2 | 2 | 2 | 2 | 2 | 2 | 2 | 2 | 2 |  |
| #13 | 22 | 2 | 2 | 2 | 2 | 2 | 2 | 2 | 2 | 2 | 2 | 2 |  |
| #14 | 22 | 2 | 2 | 2 | 2 | 2 | 2 | 2 | 2 | 2 | 2 | 2 |  |
| #15 | 22 | 2 | 2 | 2 | 2 | 2 | 2 | 2 | 2 | 2 | 2 | 2 |  |
| #16 | 22 | 2 | 2 | 2 | 2 | 2 | 2 | 2 | 2 | 2 | 2 | 2 |  |
| #17 | 22 | 2 | 2 | 2 | 2 | 2 | 2 | 2 | 2 | 2 | 2 | 2 |  |
| #18^b^ | 22 | 2 | 2 | 2 | 2 | 2 | 2 | 2 | 2 | 2 | 2 | 1 | del(Xq)^c^ |
| #19^b^ | 23 | 2 | 2 | 3 | 2 | 2 | 2 | 2 | 2 | 2 | 2 | 2 |  |
| #20^b^ | 22 | 2 | 2 | 2 | 2 | 2 | 2 | 2 | 2 | 2 | 2 | 0 | der(X)^d^ |

^a,b^Images of mFISH/FISH analysis were shown in Fig 2A (#6) and S3 Fig (#18–#20). ^c^deletion of long arm of chromosome X, ^d^derivative chromosome containing chromosome X-derived regions.
