# Supplementary material for: Novel cell lines derived from Chinese hamster kidney tissue
Source: PLoS One. 2022 Mar 31;17(3):e0266061. doi: 10.1371/journal.pone.0266061 (PMC8970510; doi:10.1371/journal.pone.0266061)
Supplement: S3 Table — (DOCX) [file pone.0266061.s003.docx]

**S3 Table. Karyotype analysis of immortalized CHK-Q cells.**

| **CHK-Q** | **Number of Chromosome [–]** | **Number of structurally normal chromosome [–]** | | | | | | | | | | | **Number of structurally abnormal chromosome [–]** | | |
| --- | --- | --- | --- | --- | --- | --- | --- | --- | --- | --- | --- | --- | --- | --- | --- |
|  |  | **1** | **2** | **3** | **4** | **5** | **6** | **7** | **8** | **9** | **10** | **X** | **del(9p)^c^** | **del(Xq)^d^** | **del(Xp)^e^** |
| #1 | 23 | 2 | 2 | 2 | 2 | 2 | 2 | 2 | 2 | 1 | 3 | 2 | 1 | 0 | 0 |
| #2 | 23 | 2 | 2 | 2 | 2 | 2 | 2 | 2 | 2 | 1 | 3 | 2 | 1 | 0 | 0 |
| #3 | 23 | 2 | 2 | 2 | 2 | 2 | 2 | 2 | 2 | 1 | 3 | 2 | 1 | 0 | 0 |
| #4 | 23 | 2 | 2 | 2 | 2 | 2 | 2 | 2 | 2 | 1 | 3 | 2 | 1 | 0 | 0 |
| #5 | 23 | 2 | 2 | 2 | 2 | 2 | 2 | 2 | 2 | 1 | 3 | 2 | 1 | 0 | 0 |
| #6 | 23 | 2 | 2 | 2 | 2 | 2 | 2 | 2 | 2 | 1 | 3 | 2 | 1 | 0 | 0 |
| #7 | 23 | 2 | 2 | 2 | 2 | 2 | 2 | 2 | 2 | 1 | 3 | 2 | 1 | 0 | 0 |
| #8 | 23 | 2 | 2 | 2 | 2 | 2 | 2 | 2 | 2 | 1 | 3 | 2 | 1 | 0 | 0 |
| #9 | 23 | 2 | 2 | 2 | 2 | 2 | 2 | 2 | 2 | 1 | 3 | 2 | 1 | 0 | 0 |
| #10 | 23 | 2 | 2 | 2 | 2 | 2 | 2 | 2 | 2 | 1 | 3 | 2 | 1 | 0 | 0 |
| #11 | 23 | 2 | 2 | 2 | 2 | 2 | 2 | 2 | 2 | 1 | 3 | 2 | 1 | 0 | 0 |
| #12 | 23 | 2 | 2 | 2 | 2 | 2 | 2 | 2 | 2 | 1 | 3 | 2 | 1 | 0 | 0 |
| #13 | 23 | 2 | 2 | 2 | 2 | 2 | 2 | 2 | 2 | 1 | 3 | 2 | 1 | 0 | 0 |
| #14 | 23 | 2 | 2 | 2 | 2 | 2 | 2 | 2 | 2 | 1 | 3 | 2 | 1 | 0 | 0 |
| #15^a^ | 23 | 2 | 2 | 2 | 2 | 2 | 2 | 2 | 2 | 1 | 3 | 2 | 1 | 0 | 0 |
| #16^b^ | 24 | 2 | 2 | 2 | 2 | 3 | 2 | 2 | 2 | 1 | 3 | 2 | 1 | 0 | 0 |
| #17^b^ | 22 | 2 | 2 | 2 | 2 | 2 | 2 | 2 | 2 | 1 | 3 | 1 | 1 | 0 | 0 |
| #18^b^ | 23 | 2 | 2 | 2 | 2 | 2 | 2 | 2 | 2 | 1 | 3 | 1 | 1 | 1 | 0 |
| #19^b^ | 24 | 2 | 2 | 2 | 2 | 2 | 2 | 2 | 2 | 1 | 3 | 1 | 1 | 1 | 1 |
| #20^b^ | 22 | 2 | 2 | 2 | 2 | 2 | 2 | 2 | 2 | 1 | 2 | 2 | 1 | 0 | 0 |

^a,b^Images of mFISH/FISH analysis were shown in Fig 2A (#15) and S4 Fig (#16–#20). ^c^deletion of short arm of chromosome 9, ^d,e^deletion of long and short arms of chromosome X, respectively.
