# Supplementary material for: Novel cell lines derived from Chinese hamster kidney tissue
Source: PLoS One. 2022 Mar 31;17(3):e0266061. doi: 10.1371/journal.pone.0266061 (PMC8970510; doi:10.1371/journal.pone.0266061)
Supplement: S4 Table — (DOCX) [file pone.0266061.s004.docx]

**S4 Table. Karyotype analysis of serum-free adapted CHK-Q_SF cells.**

| **CHK-Q_SF** | **Number of Chromosome　 [–]** | **Number of structurally normal chromosome [–]** | | | | | | | | | | | **Number of structurally abnormal chromosome [–]** | | | | **Other structurally abnormal chromosome** |
| --- | --- | --- | --- | --- | --- | --- | --- | --- | --- | --- | --- | --- | --- | --- | --- | --- | --- |
|  |  | **1** | **2** | **3** | **4** | **5** | **6** | **7** | **8** | **9** | **10** | **X** | **dup(2p)**  **der(2;4)** | **der(3;4)** | **der(6;9)** | **del(Xq)** |  |
| #1^a^ | 24 | 2 | 1 | 2 | 1 | 2 | 2 | 2 | 2 | 1 | 4 | 2 | 1 | 1 | 1 | 0 |  |
| #2 | 24 | 2 | 1 | 2 | 1 | 2 | 2 | 2 | 2 | 1 | 4 | 2 | 1 | 1 | 1 | 0 |  |
| #3 | 24 | 2 | 1 | 2 | 1 | 2 | 2 | 2 | 2 | 1 | 4 | 2 | 1 | 1 | 1 | 0 |  |
| #4 | 24 | 2 | 1 | 2 | 1 | 2 | 2 | 2 | 2 | 1 | 4 | 2 | 1 | 1 | 1 | 0 |  |
| #5 | 24 | 2 | 1 | 2 | 1 | 2 | 2 | 2 | 2 | 1 | 4 | 2 | 1 | 1 | 1 | 0 |  |
| #6 | 24 | 2 | 1 | 2 | 1 | 2 | 2 | 2 | 2 | 1 | 4 | 2 | 1 | 1 | 1 | 0 |  |
| #7 | 24 | 2 | 1 | 2 | 1 | 2 | 2 | 2 | 2 | 1 | 4 | 2 | 1 | 1 | 1 | 0 |  |
| #8 | 24 | 2 | 1 | 2 | 1 | 2 | 2 | 2 | 2 | 1 | 4 | 2 | 1 | 1 | 1 | 0 |  |
| #9^b^ | 24 | 2 | 1 | 1 | 1 | 2 | 2 | 2 | 2 | 1 | 4 | 2 | 1 | 1 | 1 | 0 | dup(3q)^c^ |
| #10 | 25 | 2 | 1 | 2 | 1 | 2 | 2 | 2 | 2 | 1 | 4 | 2 | 1 | 1 | 1 | 1 |  |
| #11^b^ | 24 | 2 | 1 | 2 | 0 | 2 | 2 | 1 | 2 | 1 | 4 | 1 | 1 | 1 | 1 | 1 | t(4p7q)^d^ |
| #12 | 24 | 2 | 1 | 2 | 1 | 2 | 2 | 2 | 2 | 1 | 4 | 1 | 1 | 1 | 1 | 1 |  |
| #13^b^ | 24 | 2 | 1 | 2 | 1 | 2 | 2 | 2 | 2 | 1 | 4 | 1 | 1 | 1 | 1 | 0 | der(X;10)^e^ |
| #14^b^ | 24 | 2 | 1 | 2 | 1 | 2 | 2 | 2 | 2 | 0 | 4 | 1 | 1 | 1 | 1 | 0 | der(3;9)^f^, der(X;3;4)^g^ |
| #15 | 25 | 2 | 1 | 2 | 1 | 2 | 2 | 2 | 2 | 1 | 4 | 3 | 1 | 1 | 1 | 0 |  |
| #16^b^ | 26 | 2 | 1 | 2 | 1 | 2 | 2 | 2 | 2 | 1 | 4 | 3 | 1 | 1 | 1 | 0 |  |
| #17 | 26 | 2 | 1 | 2 | 1 | 2 | 2 | 2 | 2 | 1 | 5 | 3 | 1 | 1 | 1 | 0 |  |
| #18^b^ | 25 | 2 | 1 | 2 | 2 | 1 | 2 | 2 | 2 | 1 | 5 | 2 | 1 | 0 | 1 | 0 | der(3;5)^h^ |
| #19 | 25 | 2 | 1 | 2 | 2 | 2 | 2 | 2 | 2 | 1 | 4 | 3 | 1 | 0 | 1 | 0 |  |
| #20^b^ | 24 | 2 | 1 | 2 | 1 | 2 | 2 | 2 | 2 | 1 | 4 | 2 | 0 | 1 | 1 | 0 | ins(2;4)^i^ |

^a,b^Images of mFISH/FISH analysis were shown in Fig 2A (#1) and S5 Fig (#9, #11, #13, #14, #16, #18, #20). ^c^duplication of long arm of chromosome 3, ^d^translocation between short arm of chromosome 4 and long arm of chromosome 7, ^e,f,g,h^derivative chromosome containing chromosome X and 10, 3 and 9, X, 3 and 4, and 3 and 5-derived regions, respectively, ^i^insertion of chromosome 4-derived regions into chromosome 2.
