# Supplementary figures and images for: Novel cell lines derived from Chinese hamster kidney tissue
Source: PLoS One. 2022 Mar 31;17(3):e0266061. doi: 10.1371/journal.pone.0266061 (PMC8970510; doi:10.1371/journal.pone.0266061)

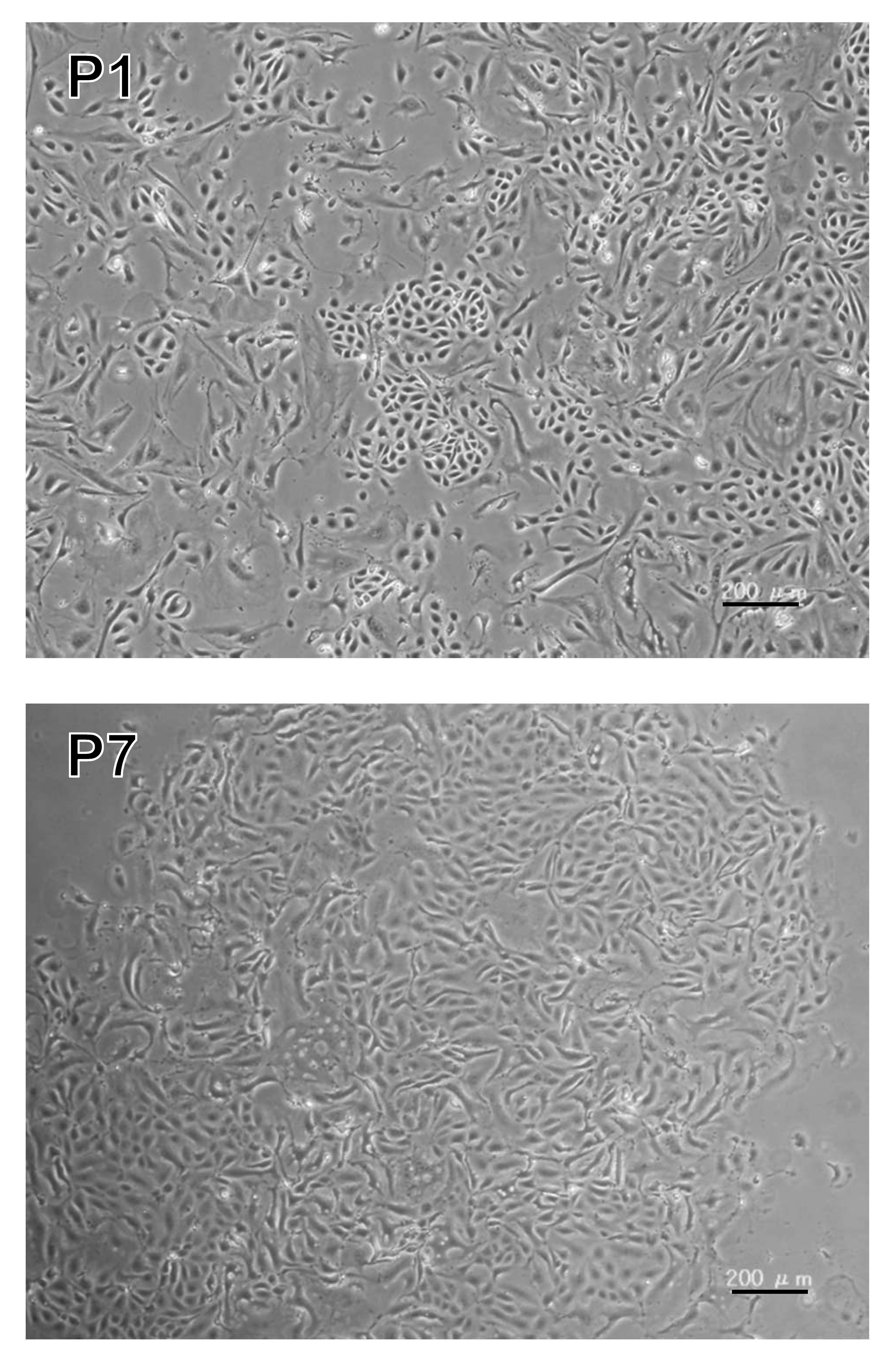

Supplement: S1 Fig — Scale bars = 200 μm. (TIF) [file pone.0266061.s005.tif]

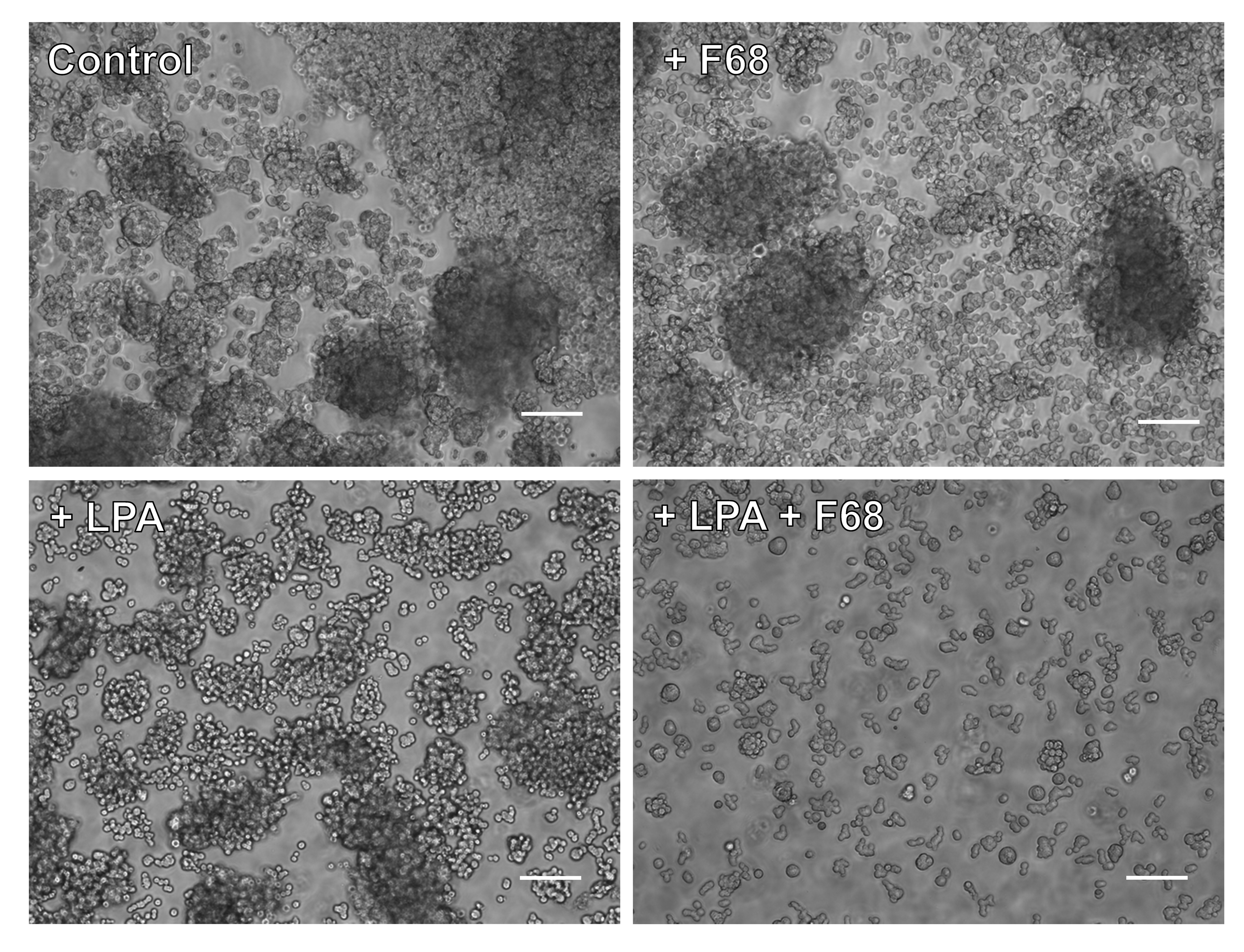

Supplement: S2 Fig — Cells were cultured in the absence (Control) or presence of 0.1% Pluronic F-68 (F68) and/or 1 μM 1-oleoyl lysophosphatidic acid (LPA). Scale bars = 100 μm. (TIF) [file pone.0266061.s006.tif]

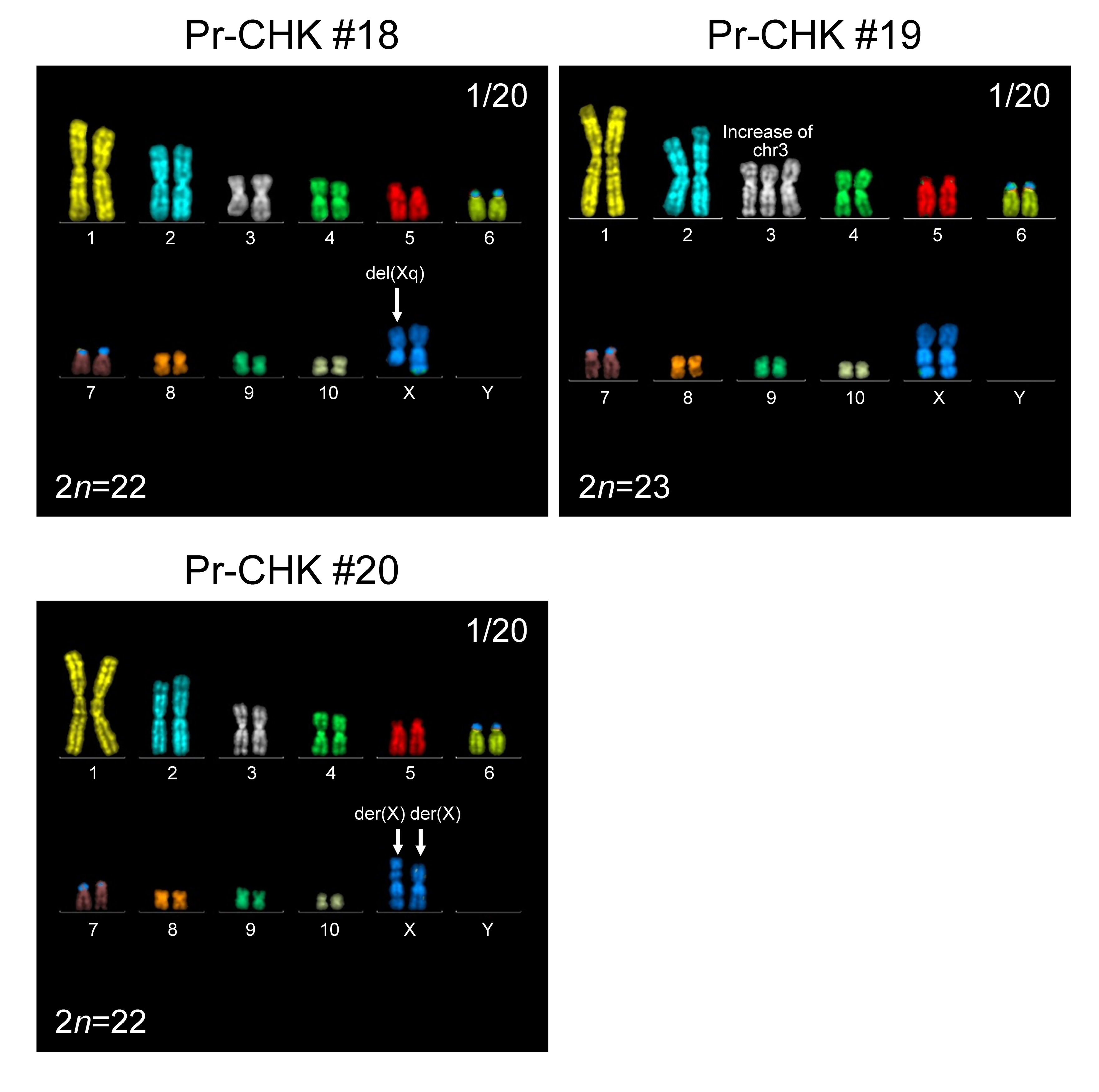

Supplement: S3 Fig — del(Xq), deletion of the long arm of X chromosome; der(X), derivative chromosome containing X chromosome-derived regions. (TIF) [file pone.0266061.s007.tif]

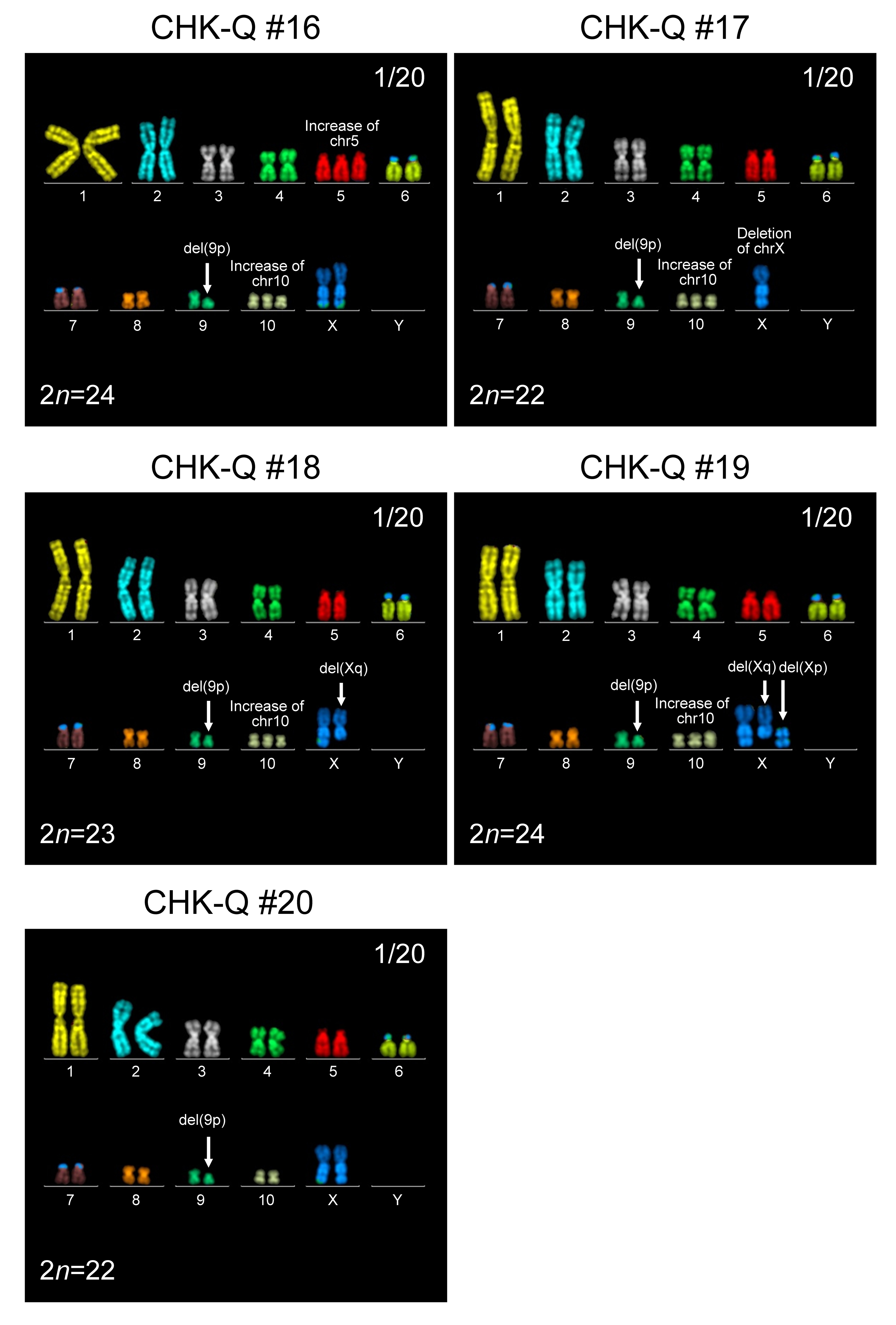

Supplement: S4 Fig — del(9p), deletion of the short arm of chromosome 9; del(Xq) and del(Xp), deletion of the long and short arms of the X chromosome, respectively. (TIF) [file pone.0266061.s008.tif]

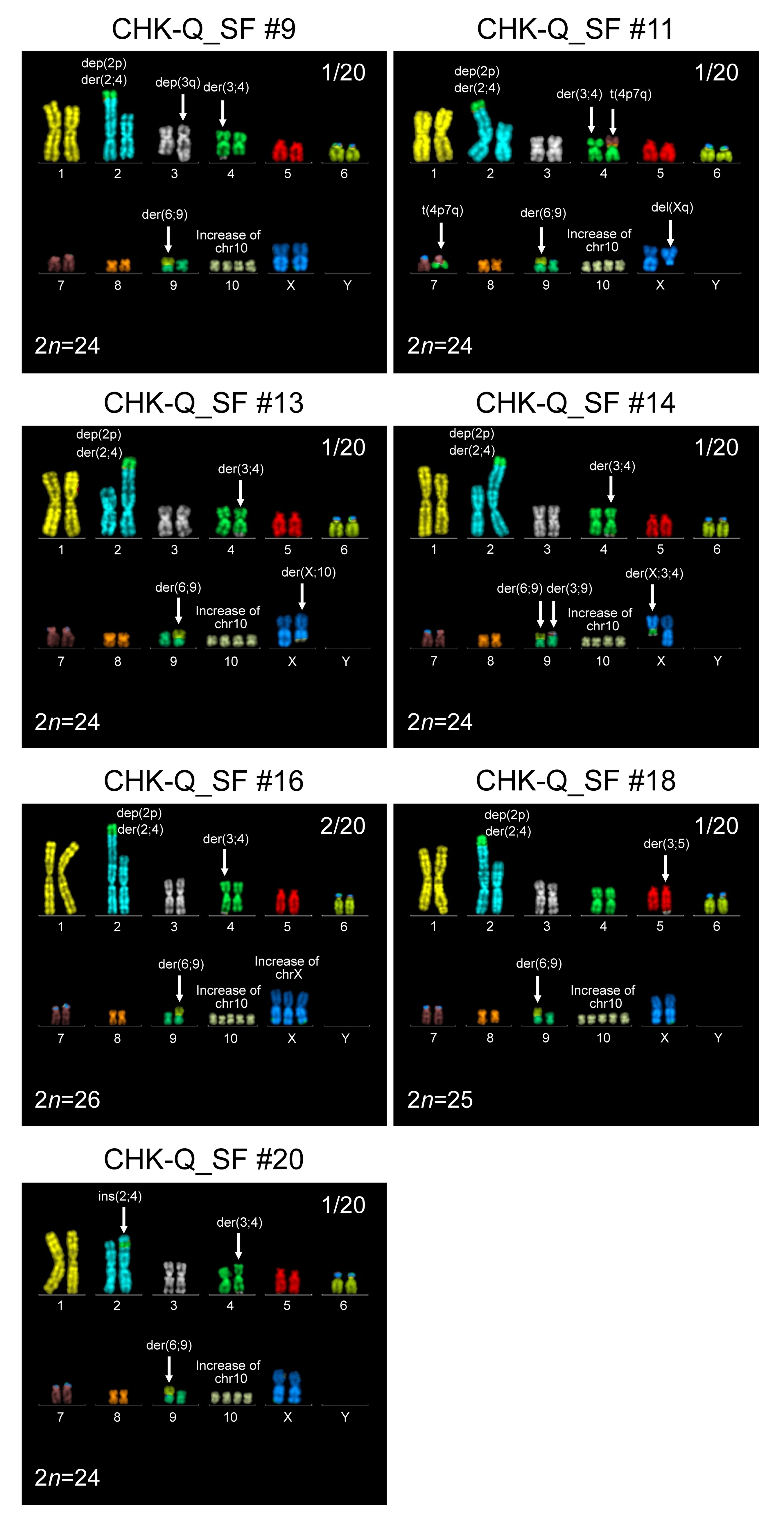

Supplement: S5 Fig — dup(3q), duplication of the long arm of chromosome 3; t(4p7q), translocation between the short arm of chromosome 4 and the long arm of chromosome 7, der(X;10), derivative chromosome containing X chromosome- and chromosome 10-derived regions; der(3;9), derivative chromosome containing chromosome 3- and 9-derived regions; der(X;3;4), derivative chromosome containing X chromosome- and chromosome 3- and 4-derived regions; der(3;5), derivative chromosome containing chromosome 3- and 5-derived regions; ins(2;4), insertion of chromosome 4-derived regions into chromosome 2. (TIF) [file pone.0266061.s009.tif]

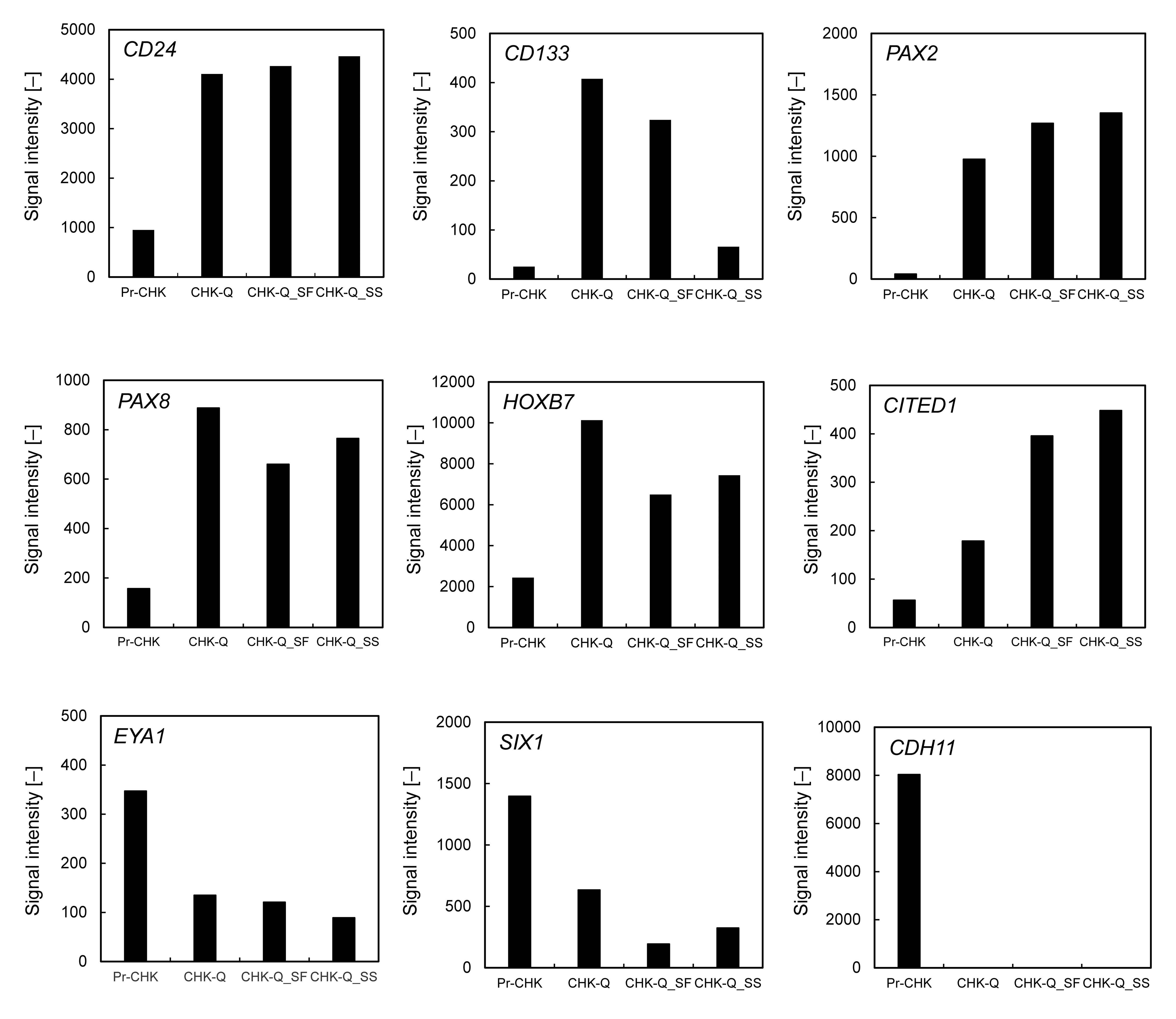

Supplement: S6 Fig — Relative gene expression of renal stem cell markers (CD24, CD133, PAX2, PAX8, HOXB7, CITED1, EYA1, SIX1, and CDH11) for Pr-CHK, CHK-Q, CHK-Q_SF and CHK-Q_SS cells. (TIF) [file pone.0266061.s010.tif]

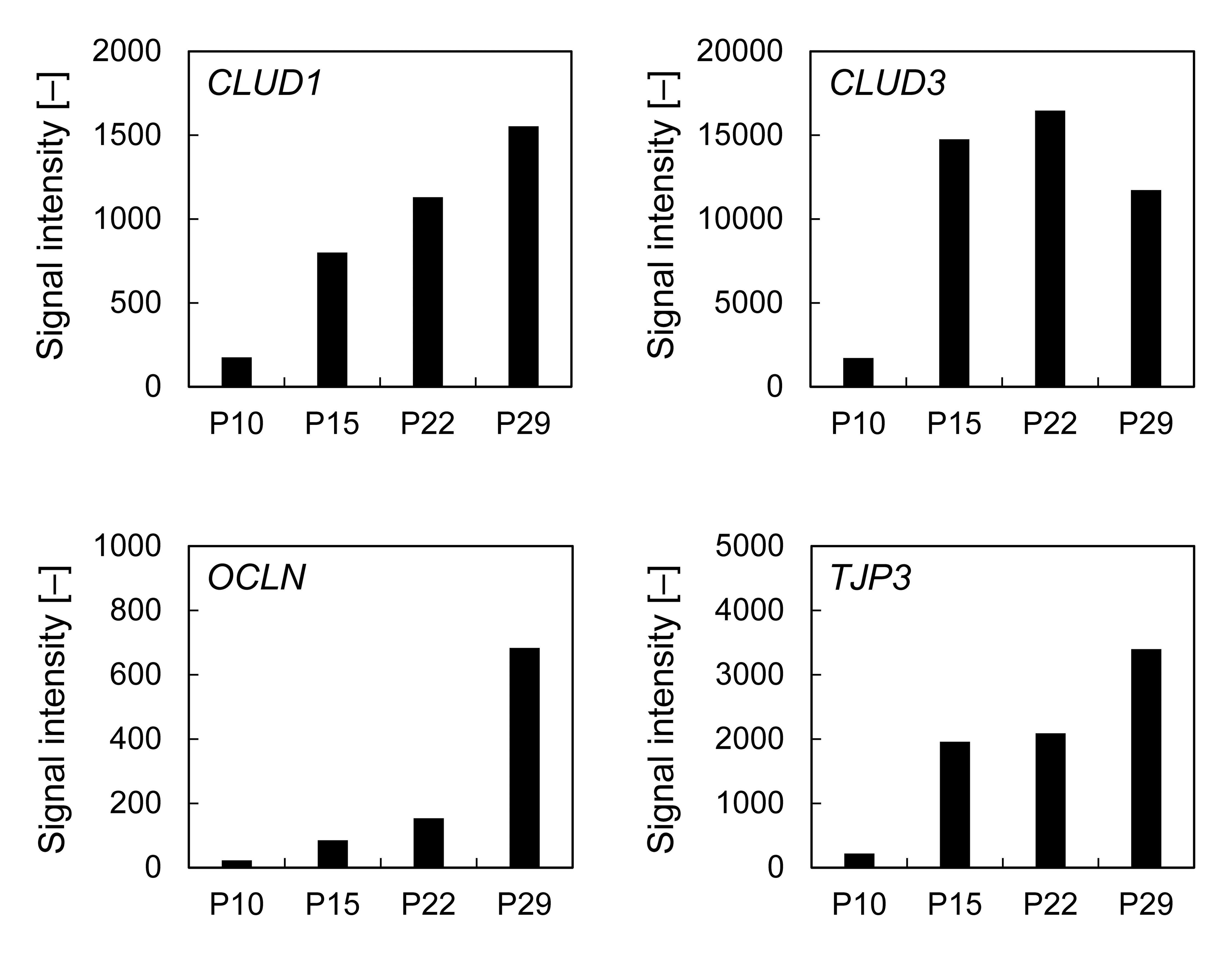

Supplement: S7 Fig — Relative gene expression of tight junction-related genes (CLUD1, CLUD3, OCLN, and TJP3) in CHK cells during culture at passages 10 (P10), 15 (P15), 22 (P22), and 29 (P29). (TIF) [file pone.0266061.s011.tif]

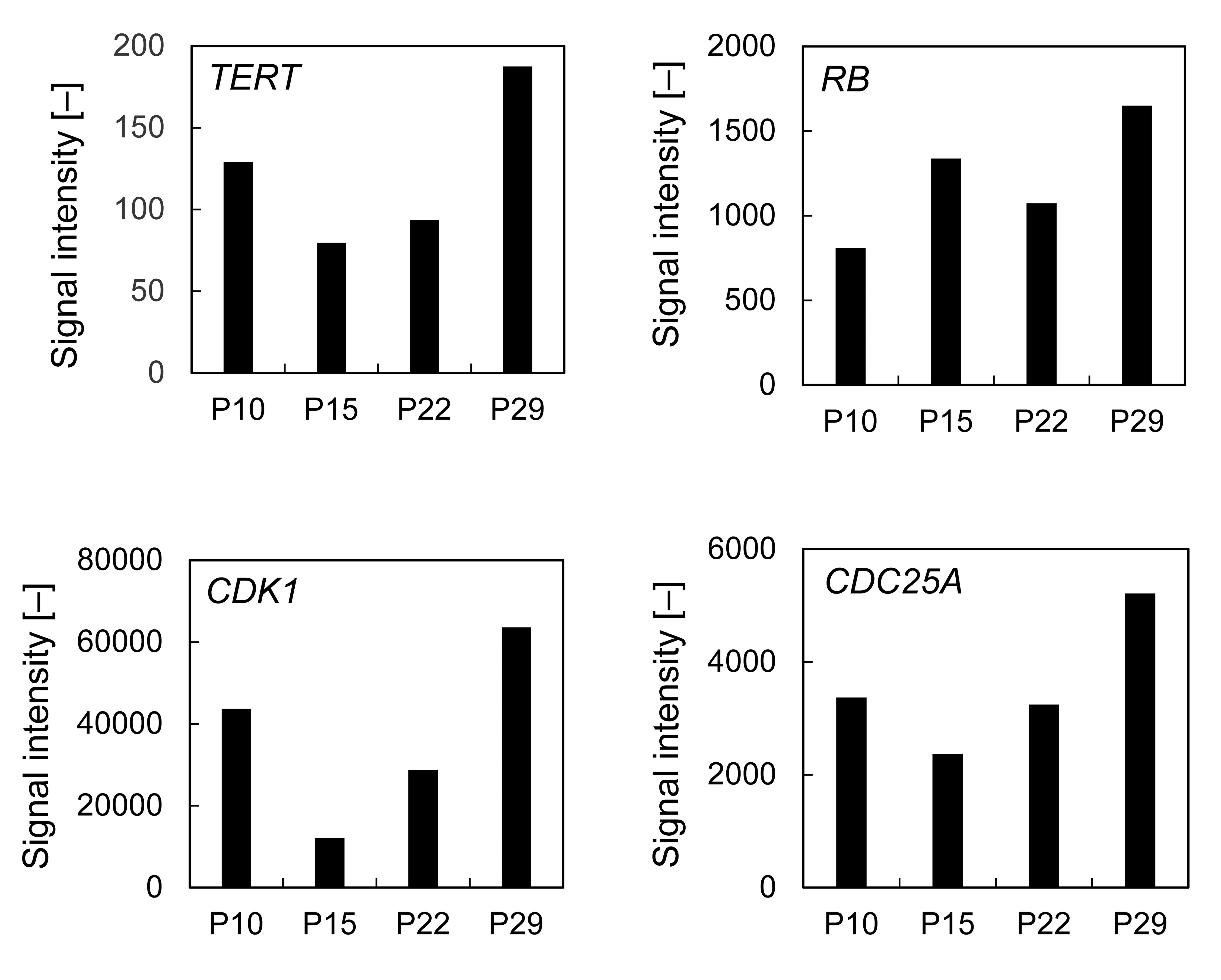

Supplement: S8 Fig — Relative gene expression of representative senescence and cell cycle-related genes (TERT, RB, CDK1, and CDCK25A) in CHK cells during culture at passages 10 (P10), 15 (P15), 22 (P22), and 29 (P29). (TIF) [file pone.0266061.s012.tif]

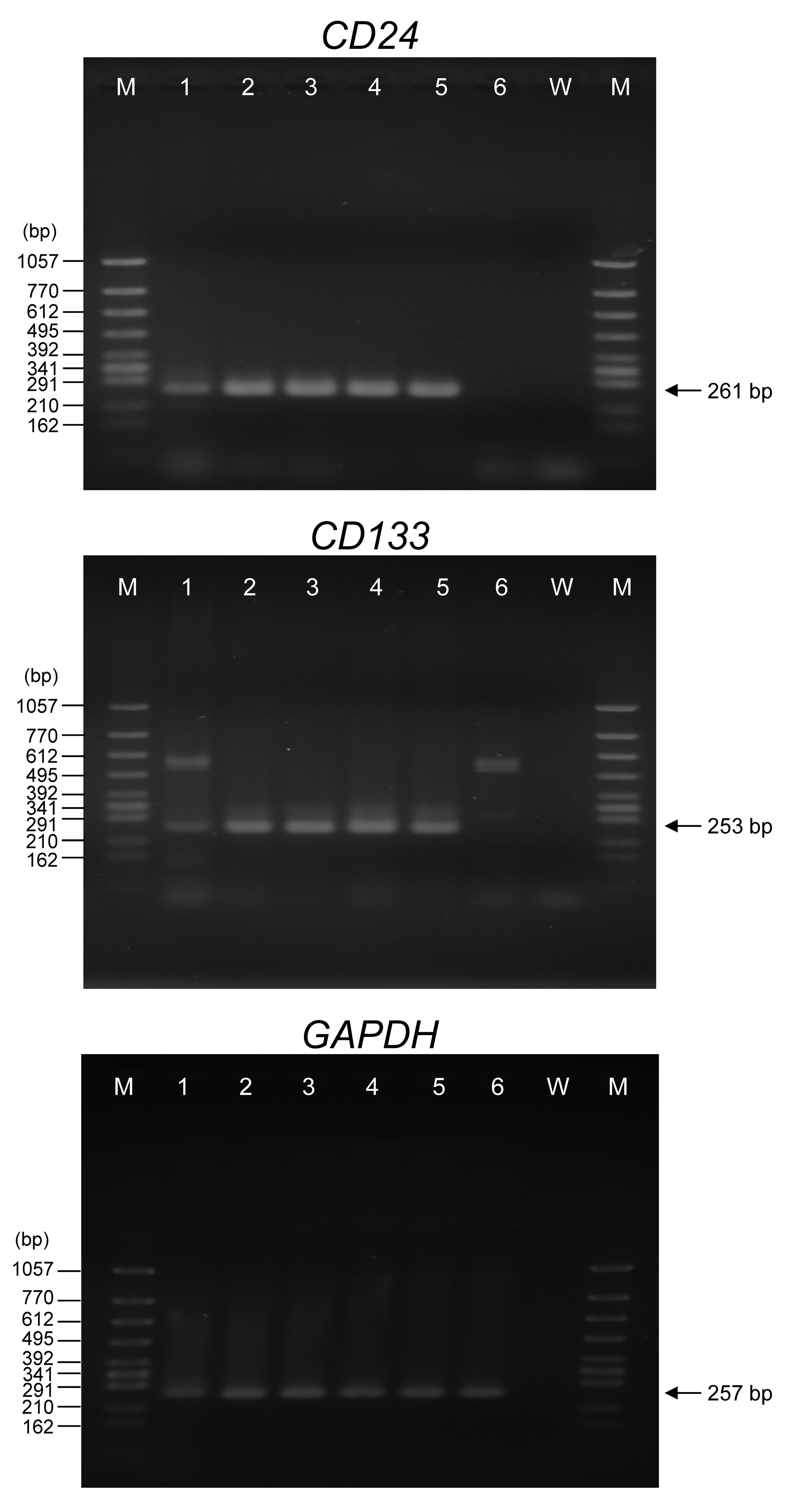

Supplement: S9 Fig — The renal stem cell marker genes (CD24 and CD133) were amplified by PCR using the primer pairs shown in S1 Table. Lane M, DNA molecular weight markers (phiX174–HincII digest); lane 1, kidney tissue cells of Chinese hamster; lane 2, pre-immortalized primary cells; lanes 3–4, two CHK-Q cell clones; lane 5, CHK-Q_SF cell clone; lane 6, CHO-K1 cells; lane W, distilled water. GAPDH was used as an internal control. (TIF) [file pone.0266061.s013.tif]

# CD24

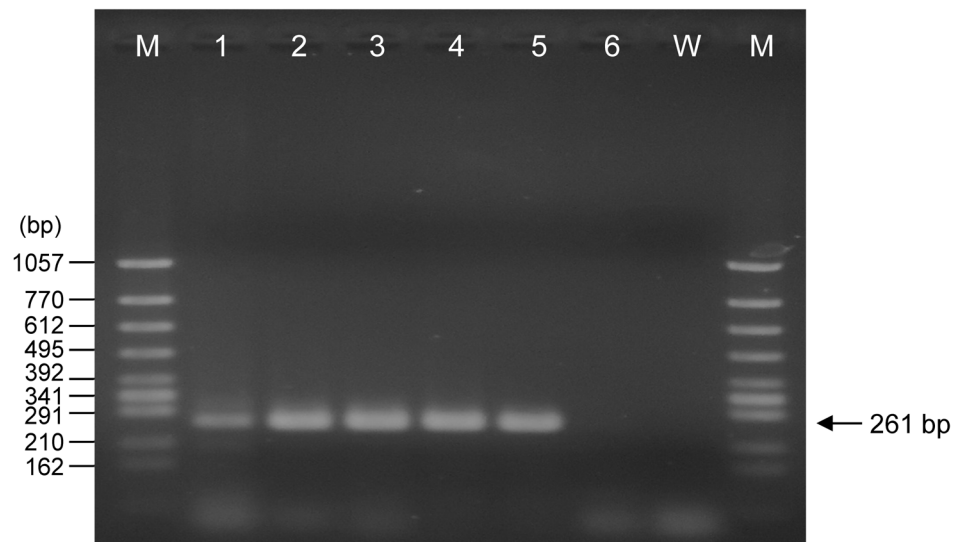

*CD133*

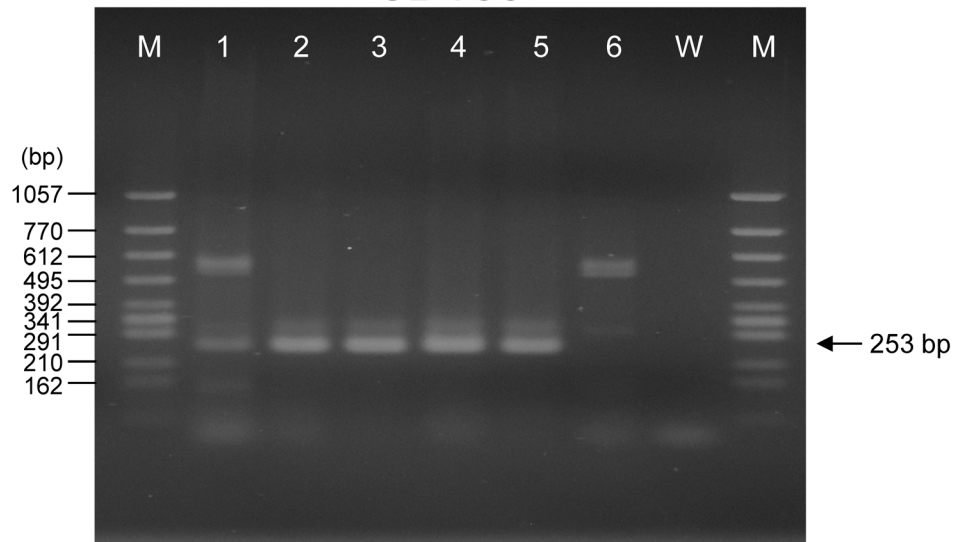

*GAPDH*

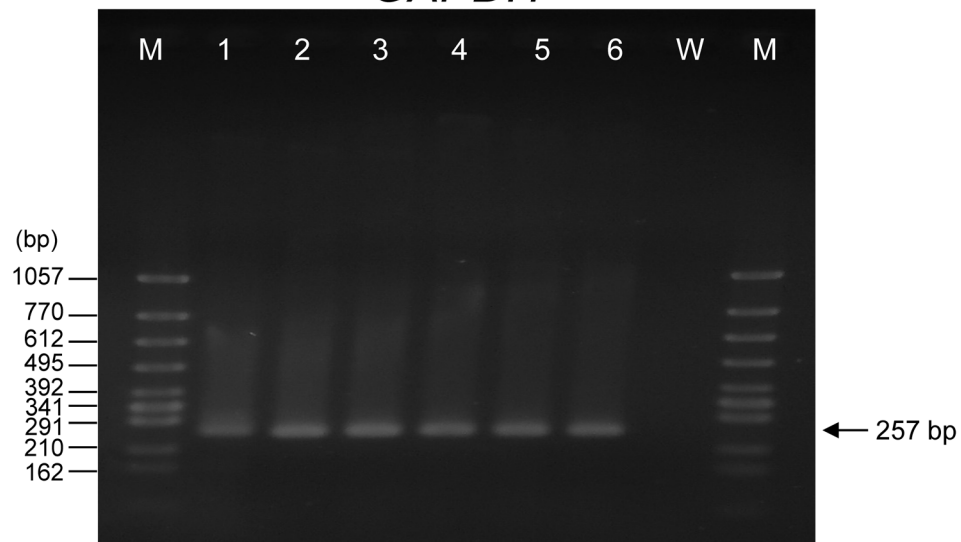

Supplement: S1 Raw images — (PDF) [file pone.0266061.s014.pdf]
